# Trust Model Concept for IoT Blockchain Applications as Part of the Digital Transformation of Metrology

**DOI:** 10.3390/s22134708

**Published:** 2022-06-22

**Authors:** Kruno Miličević, Luka Omrčen, Mirko Kohler, Ivica Lukić

**Affiliations:** Faculty of Electrical Engineering, Computer Science and IT Osijek, Josip Juraj Strossmayer University of Osijek, 31000 Osijek, Croatia; luka.omrcen@ferit.hr (L.O.); mirko.kohler@ferit.hr (M.K.); ivica.lukic@ferit.hr (I.L.)

**Keywords:** blockchain, IoT, metrology

## Abstract

Trends for the digital transformation of metrology and regulation of metrology through IT have some keywords in common with the main properties of the blockchain, such as traceability, immutability, and machine-readable documents. The possible applicability of the blockchain as an innovative IT solution for metrology regulation is known in the scientific community. Still, blockchain implementation must consider the entire metrology pyramid—the technical aspects and the legal framework intrinsic to metrology. This is also valid for possible IoT blockchain applications. In resolving the issues, this paper applies a bottom-up approach, starting from IoT devices analyzed as oracles and building up to the sole definition of measurement units, thereby discussing technical aspects concerning relevant standardization documents. The resulting trust model concept encompasses the vertical and horizontal traceability of the measurement results (oracle data), where normative standards and legal requirements are crucial for building trust. Conclusively, for practical implementations, it will be necessary to analyze blockchain properties and applicability with a view to the standard requirements, as shown for WELMEC.

## 1. Introduction

**Figure 1:**
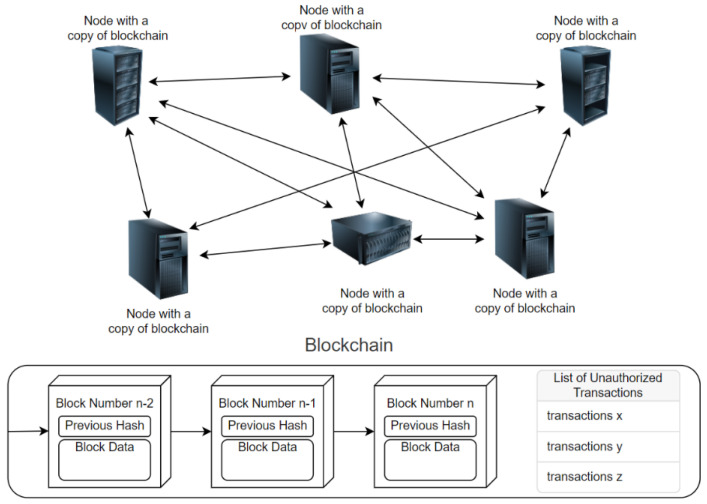
Basic blockchain structure.

Blockchain as a technology lays a foundation for Web 3.0, mainly due to its distributed nature of decision making and verifying, i.e., making any intermediaries or authorities obsolete. To establish trust in blockchain technology, it is necessary to ensure the tripod: security, authenticity, and integrity. Once the data are on the internet, we can, with reasonable certainty, be sure that the tripod is strong and secure in the case of the blockchain. Namely, the blockchain is a growing list of records (blocks) that are securely linked together: each block contains a cryptographic hash of the previous block, a timestamp, and transaction data, as shown in Figure 1. Consequently, the data in any given block cannot be tampered with without altering all subsequent blocks. Of course, it is doable, but various consensus mechanisms are defined to make it too risky and unprofitable [1,2].

To interact with blockchain in a secure way, public-key (asymmetric) cryptography is used. For example, a user publishes his public key to expose himself on the blockchain for interaction with other users, e.g., expecting to receive a cryptocurrency payment. The paying user makes a transaction using the public key as the address. The receiving user can access those assets using his private key only, which is unknown to the public.

Thus, the blockchain technology itself tackles those issues mainly successfully, but there is a critical weakness of the starting point in the case of IoT applications—to establish trust in data measured by IoT devices, or said in blockchain terminology, to verify offline data collected through so-called hardware oracles. Namely, as emphasized in [3], a recent systematic literature review on the subject, less than 10% underlined the limitations of the oracle problem. In a more detailed state-of-the-art analysis, we have made an overview of relevant references, as shown in Appendix A.

This paper’s goal is to tackle the issue of hardware oracles, with emphasis on a holistic approach typical for metrology hierarchy, starting from the comparison and synergy of blockchain technology and the digital transformation of metrology in Section 2. Section 3 shows oracle types and their relevance for IoT applications. This paper’s main contribution is presented in Section 3, where we show oracle peculiarities for IoT blockchain applications, and in Section 4, which defines the trust model concept for IoT blockchain, starting from the IoT device level, where we analyze the possibility of implementing a WELMEC standard using the blockchain, and go through levels of metrology hierarchy up to the sole definition of the measurement unit. This paper, in general, shows how to integrate IoT architecture into the blockchain (and vice versa), but according to the already existing and globally accepted metrology hierarchy. Section 5 summarizes the main conclusions.

## 2. Blockchain as a Technical Solution for the Digital Transformation of Metrology

If we go back to basics and look beyond the buzzwords, the oracle problem boils down to the problem of trust in measured physical quantity—a problem well known for centuries and targeted by metrology. Modern metrology as a scientific discipline has its roots in Metre Convention established in 1795. The following important years were 1960, the year of the creation of the International System of Units (SI), and 2019, when four SI base units were redefined.

In all these years, the master premise for establishing trust in measurement results was (metrological) traceability—the property of a measurement result whereby the result can be related to a reference through a documented unbroken chain of calibrations, each contributing to the measurement uncertainty [4,5].

To establish trust in the data chain from an IoT device through a communication channel to the user, we can apply the same metrological principles and rely on well-defined metrology standards and procedures in doing so. It is a lucky coincidence, or, more accurately, a consequence of technological development, especially in the IT domain, that metrology is going through the process of digital transformation itself [6,7,8].

According to [9], the digital transformation of metrology should provide technical solutions to the following trends:

- The move to an increasingly paperless world, including reduced use of paper money;
- Continued introduction of digitization in all areas;
- The redefinition of the SI being likely to lead to increased availability of intrinsic standards;
- The IoT leading to increased size and complexity in measuring systems, with a proliferation of sensors; and
- Artificial intelligence becoming an increasingly important feature in the software of measuring instruments.

According to [9], the digital transformation of metrology requires a holistic approach that includes all relevant aspects and activities—(re)calibration, (re)testing, (re)certification, (re)verification/inspection, market surveillance, accreditation, and standardization—and this holistic approach should be applied to IoT-related challenges as well in order to establish trust in IoT data and widen IoT applicability and interoperability. Thereby, the so-called FAIR+T approach is recommended for the data—data should be: findable, accessible, interoperable, re-usable, and traceable.

Regarding the blockchain IoT applications, there are numerous technical solutions discussed [10,11], also including possible IIoT applications [12]. Thereby, it seems that blockchain technology has needed properties for the FAIR+T approach, as shown in Table 1, to answer the most technical challenges of digital transformation of metrology.

Although not requested by digital transformation, there are also smart contracts, as an additional inherent mechanism in the blockchain from which measurement traceability could profit. Smart contracts were first introduced and popularized mainly through Ethereum [16,17] within the blockchain boom. Smart contracts are on blockchain-distributed computer programs, which are executed automatically when the defined terms and conditions are fulfilled. 

This functionality is beneficial for numerous different areas [18,19], but the literal understanding of “smart contracts” as “contracts” is problematic because, in the case of a dispute, courts might rely on the underlying intent of the parties rather than the written code [18]. However, in metrology, smart contracts could be used for automated decision making, e.g., about (un)successful calibration process of an IoT device, based on exact values with clear thresholds, and for recording the decision on the blockchain in the form of a digital calibration certificate (DCC) [20,21]. In doing so, the trust in measured values, i.e., in oracles (IoT devices) as inputs for smart contracts has a crucial role.

## 3. Oracle Types and Relevance for IoT Applications

**Figure 2:**
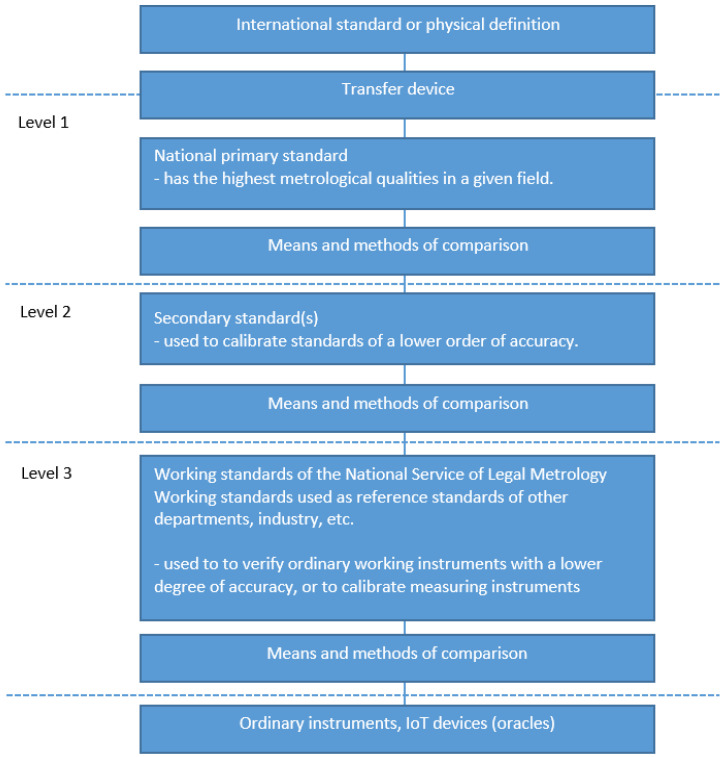
National hierarchy scheme and role of IoT device [22].

The needed holistic approach is imperative for applying blockchain to the whole metrology pyramid. Because the measurement results are the foundation of the pyramid, in analyzing the applicability of blockchain, we will take a bottom-up approach, i.e., start with the IoT devices as measuring instruments (MI) and work up the pyramid. Thereby, we will follow the hierarchy, i.e., the first three levels, defined in [22], as shown in Figure 2.

According to [22], there are general rules for establishing hierarchy schemes for measuring instruments. The primary purpose of these hierarchies is to establish trust in measurement results and consequently in products and services based on them. This should also be the priority for IoT devices as blockchain oracles.

The problem of confidence in the measured results at the level of IoT devices within blockchain technology comes down to the so-called oracle problem [23,24]. Therefore, the terms “oracle” and “IoT device” will be used interchangeably. 

In the blockchain ecosystem, oracles are trustable entities that feed the blockchain network with information from the external world. In the context of metrology, the oracles are IoT devices connected to the blockchain on one side and compliant with the metrology regulations on the other [24].

In general, oracles take on several key functions [25], but in the case of metrology/IoT applications, the most relevant are:

- Monitoring the blockchain network to check for incoming user or smart contract requests for measured data.
- Performing some type of computation, such as calculating a median or more complex parameters from multiple oracle submissions (e.g., extended Kalman filter, see Section 3.5), and calculating a critical value for threshold defined in the calibration procedure.
- Verifying (sign) and sending measured (or calculated) data to the blockchain for processing by the smart contract.

There are several relevant options and parameters for oracles, as shown in the following paragraphs, where we also emphasize the peculiarities of IoT applications.

### 3.1. Origins of Data That Oracles Provide to Blockchain-Based Applications

Data that oracles provide to the blockchain are [23]:

- Web content;
- Sensor data.

It is clear that for IoT applications, the sensor data are dominant. However, it is also possible for oracles to provide web content, e.g., data about users and details of the certificate documents.

### 3.2. Types of Oracles for Use in Blockchain-Based Applications Regarding the Input/Output

Types of oracles depend on the input/output and their role [23]:

- Software oracles—oracles that provide online information to the blockchain, e.g., additional data about the calibration document and laboratory.
- Hardware oracles—oracles that provide information from physical devices, in our case from IoT device, to the blockchain. According to the previous point (Section 3.1), i.e., dominant sensor data, it is expected that hardware oracles have higher usage in IoT applications than software oracles.
- Inbound oracles—oracles that provide smart contracts with data from the external world, e.g., from accreditation institutions.
- Outbound oracles—oracles that send information to the outside world, e.g., to users interested in measurement traceability.
- Consensus-based oracles—data passed to the blockchain are treated as a result of a consensus of multiple oracles, e.g., if it is required to decide about data based on multiple hardware oracles (sensors).

### 3.3. Number of Sources That Are Used by Oracles

Depending on the number of sensors, the oracles can be:

- One sensor -> single-source oracle;
- Multiple sensors -> multiple-source oracle.

The application and the needed level of trust determine which approach has to be used. Of course, the single-source oracle is, in general, more often needed and more straightforward to implement. However, multiple-source oracles are required if one wants to decide based on multiple sensors, aggregated data, or needs data from multiple sensors to raise trust or ensure redundancy.

Thereby, as described in Section 3.5, it is recommended to use different sensor types to ensure data credibility.

### 3.4. Validation of the Data That Oracles Provide to Blockchain-Based Applications

The main idea and driving force for the blockchain is the verification of data that do not need any intermediaries or authority in general but is based on a consensus reached among the blockchain users themselves. For example, the goal of Bitcoin, as the first and the most prominent blockchain application, was to enable online payments directly without going through a financial institution [1].

Although the applicability of the blockchain as electronic cash is becoming doubtful and there are some pending challenges in general, e.g., related to security and performance [26], the blockchain’s main inherent specificity, compared to centralized systems, is the distributed consensus-based verification, which avoids the central authority. The most prominent consensus types are Proof of Work (PoW), Proof of Stake (PoS) and Proof of Authority (PoA) [2,27]. There are numerous alternative approaches, but in general, the main goal of consensus is, as already mentioned in the Introduction, to attempt to make fraud too risky and unprofitable for the verifier. For example, PoW verifiers invest high amounts of computing power and, in general, it is not profitable to invest it for false verification [18]. On the other hand, in PoS, the blockchain tokens are invested as a stake, i.e., the verifiers risk losing them if they verify incorrect data (transactions).

PoA somewhat deviates from this logic. Namely, the PoA is a consensus method that allows a designated number of blockchain actors to validate transactions, limiting the idea of a broad consensus. Thereby, the actors are staking their identity, i.e., if they undertake some malicious activities, their identity would be disclosed and, consequently, their reputation ruined, as well as their possible future active role in reaching consensus, i.e., validating blockchain transactions.

Due to the strict hierarchical structure of metrology processes with clear authorities, it is natural to use the PoA for measurement results. However, the blockchain loses distributed consensus-based verification as its main comparative advantage. Nevertheless, the blockchain keeps its other specific properties, such as distributed and immutable data storage. Thus, the decision about the consensus type should be made regarding specific applications, considering their requirements. For instance, the Proof-of-Reputation could also be an alternative [2].

### 3.5. Security of Data Sent by Oracles

Encryption is essential for ensuring the security of transferred data and the authentication of users. In brief, the blockchain infrastructure can [9,14,15]:

- Provide integrity, authenticity, and non-repudiation of legally relevant (LR) information [28];
- Store and attest public keys from IoT devices and all other participants;
- Avoid a trusted-third-party cost with digital certificates;
- Provide a solution that does not depend on a trusted third party.

Additionally, the security of measuring devices (oracles) must be ensured using secure IoT communication protocols [29,30] and anti-tampering protection.

Namely, to guarantee that oracles will sense and measure (record) true values of the measured quantity, it is necessary to minimize the possibilities of tampering. The tampering could happen on three different levels:

- Tampering oracle software—software tampering could be mitigated by creating a hash based on software code, i.e., any unauthorized change of code would be detected by unmatched hashes. However, there is also a possibility for an authorized change of code, e.g., through updates carried out online. To allow it, it is needed to have a public key infrastructure (PKI), so that each oracle (and its code) can be accessed online if the user has a corresponding key [14,15]. Thereby, it is needed to follow relevant standards as well. For instance, WELMEC [28] distinguishes legally relevant (LR) and legally non-relevant software (LNR). Of course, LR is the main target to be protected from unauthorized changes, but the protection of LNR could also raise the trust in measurement results.
- Tampering oracle hardware—anti-tampering techniques are generally divided into four categories: tamper prevention, tamper detection, tamper response and tamper evidence. They include various methods and safety mechanisms such as encapsulation and coating of the hardware device [31], anti-tamper switches, sensors and circuitry [32], unique hardware properties of the device [33], secure cryptographic processors and device boot procedure that is designed specifically to detect tampering that has occurred while the oracle has been without power supply [34].
- Tampering sensor input—the most challenging issue is detecting the tampering of sensor input. For instance, if we want to know the temperature of a warehouse, truck trailer in transport, etc., the question is how we can be sure that the sensor is not maliciously placed in a temperature-controlled location that is isolated from the location intended for measurements. The solution could be based on consensus-based oracles (see Section 3.2). However, using multiple sensors of the same type (e.g., classical temperature sensors) is not a practical solution because they can be simply manipulated in the same way as single sensors. A better approach would be combining different sensor types, which would complicate possible malicious manipulation, for instance, classical temperature sensors combined with computer vision to detect a change in sensor surroundings and with infrared cameras as additional sources for temperature data on surrounding surfaces. The final estimation of the measured value could be completed using extended Kalman filters or some other method to combine data from different sensors [35,36]. In this way, the in situ inspections of measuring instruments and field surveillance [22] could be replaced by remote checks via blockchain smart contracts. Of course, this kind of system would be fairly complicated, and it is important to analyze for which possible applications it could be cost-effective.

## 4. Trust Model Concept for IoT Blockchain

### 4.1. Applying the Blockchain Technology to the IoT Device Level

To ensure trust in the IoT device (measuring instrument) as a blockchain oracle, it is necessary to rely on corresponding standards, emphasizing the device itself as an origin of trust. Of course, a set of standards depends on particular device and built-in sensor. To present the concept of applying a device-related standard in the context of the blockchain, we will analyze the WELMEC standard for determining software risk categories in a measuring device [28]. Table A2 in Appendix B shows Ethereum and Hyperledger Fabric [16] blockchain platforms that can be integrated for metrology applications and whether they can be used to determine software risk categories according to WELMEC categories. Conclusions made for Ethereum are applicable for some other platforms, such as Cardano or Solana [37,38]. 

The following WELMEC requirement sets that are relevant and covered in the table analysis are: long-term storage of measurement data (L), the transmission of measurement data (T), software download (D), and software separation (S). Each set of these requirements is only applicable if they have a corresponding function.

According to Table A2 in Appendix B, blockchain technology meets the majority of WELMEC’s risk assessment standards because of its inherent qualities such as data distributivity, security, and integrity. The second part of the requirement was met through the concept of smart contracts. On the other hand, blockchain technology does not meet all WELMEC requirements. L2 and L7 do not fit into the underlying concept of blockchain technology since data must obtain a consensus among network members to be stored within a network. Furthermore, once data are finalized on the blockchain network, they can no longer be changed or lost. This exception can be made by a central authority using permissioned blockchains such as Hyperledger fabric, but it may jeopardize the integrity of the data stored within the blockchain network (L3).

**Figure 3:**
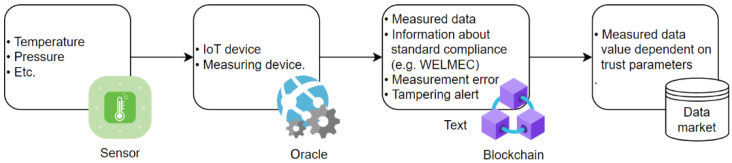
Information about measured data relevant for a data market.

To ensure scalability, it is recommended to provide data users with information about each individual property (max. 23 properties according to Table A2 in Appendix B). In this way, corresponding measured data have a higher or lower trust level. Additionally, the IoT device can provide information about its measurement error and alerts if a hardware or software tampering has occurred, as shown in Figure 3 (see Section 3.5).

All these values can be used as indicators for measured data trust and its value on potential data market as an estimation made by data seller/buyer or automatically by a smart contract which has clear defined thresholds for each of parameters, e.g., to remove the measured data from the market if a tampering event happened. The topic of a possible data market is also a significant one in the context of IoT data, but due to the length of this paper, it will not be analyzed here. However, interested readers can find more information in the literature [39,40,41].

### 4.2. Establishing the Complete Trust Hierarchy

**Figure 4:**
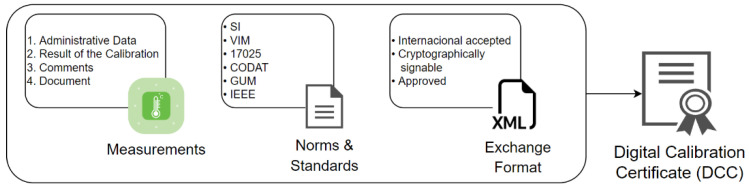
Structure of digital calibration certificates according to [42].

The IoT device as the origin of trust is essential as the starting point, but the blockchain should ensure traceability of measurement results from the sensors up to the definition of the measurement unit, through all three levels [22], providing insight into the calibration documents (certificates) as links between adjacent levels. The documents can be available online and fetched through software oracles to relieve the ledger of the data amount. Thereby, the PKI and digital signatures must be used to ensure authenticity [14,15]. Nowadays, there are several competing DCC formats, as shown in Figure 4 [20,42,43], and time will tell which one will prevail and be accepted by the metrology community and institutions. The finally embraced format will not have an impact on possible blockchain applicability. Namely, the blockchain task is to record the existence of a certificate (which must be validated by issuing institutions), and the certificate format is not crucial.

However, within the blockchain, the calibration process could be additionally automatized by using the calibrating instruments as oracles and implementing the allowed error levels (and resulting decisions) as a smart contract code. However, several peculiarities should be taken into account. Namely, contrary to the MIs on-site (Section 4.1), the instruments at levels 3 to 1 are used for calibration in a laboratory environment. Moreover, accredited laboratories carry out the measurements, resulting in a higher level of trust. Consequently, for the laboratory environment, anti-tampering solutions described in Section 3.5 are welcomed but not necessary in such a strict form, e.g., there is no need for consensus-based (multi-sensory) oracles. Furthermore, PoA can be used as a consensus method; there is no need for PoR or some other, even more complex algorithm.

**Figure 5:**
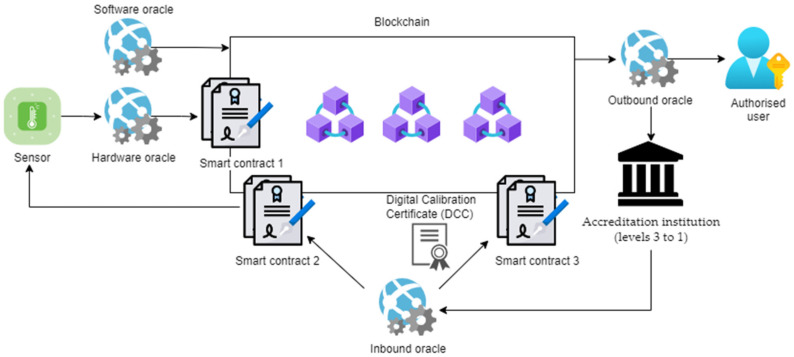
Concept of complete trust hierarchy.

The final implementation of blockchain through all levels could give an additional boost to the transparency of measurement results and resulting trust. Each measurement result could be immediately linked to measurement results at higher (calibration) levels, up to the definition of the measurement unit, as shown in Figure 5:

- Sensor—single sensor (or multi-sensor data as a result of extended Kalman filters [35,36])
- IoT device as hardware oracle provides measured data to the blockchain. Due to its possible large amount, data can be recorded off-chain and the blockchain stores just a hash as proof of data content.
- Blockchain—as recommended in Table 1, the blockchain considered for metrology-related applications is in general private, ensuring who can have access to data and write to the blockchain. However, it is not excluded that one could use also a hybrid blockchain in cases when data owner (or generator) wants parts of the data to be publicly visible, or when the scope of users is very broad, e.g., in use-cases for supply chains with high number of participants. A consortium blockchain could be an option for the trust model that is administrated by more entities, e.g., for inter-NMI applications. Who can be a verifying blockchain node and the process of authorizing a node depends on the blockchain type, but in any case, it is administered by one entity (in private and hybrid blockchain) or more entities (in consortium blockchain). Consequently, public blockchain is more limited regarding the possible use-cases for IoT blockchain applications.
- Software oracle provides additional data about oracles, users and institutions, e.g., links to oracle datasheets, general information about a laboratory, and NMI.
- Inbound oracle provides data to smart contracts to institutions for sensor verification (smart contract 2). On the other side, it provides data to smart contracts for triggering sensor recalibration (smart contract 3).
- Outbound oracle provides data to authorized users (according to their access levels).
- Smart contract 1 provides the blockchain data about IoT device (hardware oracle) WELMEC compliance and possible tampering events, triggering corresponding events, e.g., rejection of data.
- Smart contract 2 triggers sensor (re)calibration based on inbound oracle, i.e., based on digital calibration certificate issued by institutions.
- Smart contract 3 triggers (re)certification procedure carried out by accreditation institution (issuing digital calibration certificate and recording it on a blockchain).
- Digital calibration certificate issued by an accreditation institution and recorded on a blockchain.

In addition to this vertical traceability, it is also possible to show horizontal traceability, i.e., results of previous calibration periods. Although legally not so relevant as the vertical one, horizontal traceability could be important from a transparency point of view. It gives the final user information about the calibration history and maintenance of the measuring instrument.

All this information about vertical and horizontal traceability, including the information about measured data, as shown in Figure 3, is relevant for the value of measured data if offered in a data market (see Section 4.1). Thereby, the amount and type of fulfilled standards are important as well, where we can distinguish different levels of legal strictness (Table 2) between purely voluntary standards and various forms of technical regulations, which have legal relevance. There is a set of laws independent of a standard and their implementation within the trust model concept is, in general, not possible, due to its lesser level of logical unambiguity [18]. Thus, the trust model concept primarily envelops five out of six normative standards and legal requirements (Table 2).

There is some progress in applying the blockchain for levels 3 to 1, e.g., for the inter-NMI blockchain network [14], and also at the level of measuring instruments, e.g., in evaluating LR software in U-type instruments [45]. However, to fully accept blockchain technology, it is necessary to completely adapt the legal framework [13].

If we invert the procedure, i.e., try to adapt the blockchain solution to the legal framework, it is necessary to comply with relevant standards and regulations. Government institutions or international organizations define legal control of MI and type approval, including paperwork and code inspection, validation and verification, and metrological supervision, which includes quality, market, and field monitoring [6,46]. For software-controlled MI design, deployment, and inspection, refs. [28,47] are the most widely used standards. All software modules that contribute to or influence measurement findings are legally relevant, according to [28]. This covers not only the software modules that generate and process measurement data, such as oracles, but also underlying blockchain technology for transferring, storing, and representing generated MI data.

## 5. Conclusions

The blockchain has several properties that correspond to the digital transformation needs of metrology. However, trust in measurement results requires a holistic approach—there must not be any weak link in the traceability chain. Thus, no partial solutions are beneficial in the long term.

To achieve a long-term self-sustainable solution, it is necessary to synchronize the blockchain applications and development with the process of digital transformation of metrology. Thereby, the solution has two levels: the IoT device level (oracle) and the level of traceability. To build trust in the IoT device itself, it is recommended to use usual normative standards and legal requirements, as shown in the WELMEC example. For traceability level, an active role of accredited laboratories and NMIs is required. For both levels there is a possibility to automatize procedures using smart contracts, as presented in the concept of a complete trust hierarchy.

In both cases, there are several critical points for possible future research directions:

- The legal framework must be changed to legalize blockchain usage in metrology procedures.
- In order to be completely accepted and widely used, the blockchain-based trust concept in metrology must be legally mandatory. In contrast, i.e., just as an alternative in addition to the well-established tradition of the paperwork, the blockchain-based trust concept would not prevail due to the conservative nature of metrology.
- The concept, as well as its building blocks (for example, what kind of DCC format should be used) must be also adopted by the users. In doing so, the question remains of should the adoption be pushed top-down (i.e., starting from defined laws and regulations and applying them in practice) or bottom-up (i.e., waiting for which concept and elements will be accepted by the user community and then define laws and regulations also corresponding to user habits).
- Harmonization between the legal framework and technical capabilities (and limitations) of the blockchain, e.g., evaluating the content of smart contracts in comparison to the legal documents and resolving possible disputes in case of later identified discrepancies.
- Should such IoT devices as oracles communicate directly with the blockchain or should more IoT devices be connected to the internet via a gateway. In the first case, the IoT device is more costly due to its higher hardware and software complexity, but the lower number of communication intermediaries (gateways) in such kind of structure increases the data security.
- In terms of possible applications, data recorded on the blockchain contribute to the transparency and traceability, e.g., in supply chains, which raises trust in corresponding products and also their value. However, it is needed to further explore the possibilities for measured data itself to become a product, i.e., an object of trade in the data market. Additionally, in this case, the trust model is again very important, because more trust in the measured data means a higher price of the data as a product.

Due to the importance of metrology in industry, economy and everyday life in general, it is not expected that the legal framework will change soon. In the meantime, a sound basis for further changes is the development of possible technical solutions, the adaptation of standardization documents and various initiatives and discussions driven by international metrology associations and scientific communities. This paper aims to be one step in this direction.

In the future, the authors will focus on integrating IoT devices into blockchain protocols as oracles according to WELMEC regulations. The research will also include possible multi-sensor solutions to reach full trust in quantities measured via oracles. A further research direction is possible tokenization of measured data in order to offer it on a data market, where the different extent of IoT standard compliance, verified on a blockchain, will impact the measured data market value. 

## Figures and Tables

**Table 1:** Blockchain as an answer for digital representation in metrology processes.

Requirements for Digital Representation in Metrology Processes [5,9]	Blockchain Properties	Recommendations/Possible Issues
Contain all relevant information for conformity assessment, verification, market surveillance in a machine-readable way	Data comprised in transactions	The amount of data could be a problem. It is needed to use/store data in databases outside the blockchain
Contain all relevant information for customers to gain trust and confidence in the products and quality measures		
Know the relevant standards and regulations, and provide machine-readable information about it	Blockchain uses machine-readable information only	It is necessary to make relevant standards and regulations also machine-readable
Provide machine-readable interfaces for users and manufacturers to enable “smart quality assurance”		-
Combine machine-readable documents and certificates, enable automation of digital QI processes		
Be secured and validated to provide access to information only to eligible parties	Blockchain uses asymmetric cryptography to grant access to users	To limit who can have access, a private blockchain network is recommended [13,14,15]
Not requested, but it could be an additional benefit	Smart contracts embed terms and conditions of a contract between two or more parties [16,17,18]	Automated decision making and recording of the decision on the blockchain

**Table 2:** Normative standards and legal requirements according to [44].

Standard-Based Requirements (Scope of the Trust Model)					
Voluntary Standards					
		Technical Regulations			
No link with legal requirements	Can be taken into account by the courts, e.g., WELMEC 7.2 Software Guide	Conformity is a guarantee, but not the only way that requirements have been met	Conformity required by law, e.g., ISO/IEC 17025—Testing and calibration laboratories	Law based on a standard	Laws independent on a standard, e.g., national metrology laws

## Abbreviations

IoT	Internet of Things
WELMEC	(Western) European Legal Metrology Cooperation
SI Units	International System of Units
FAIR+T	Findable, Accessible, Interoperable, Re-usable, and Traceable
IIoT	Industrial Internet of Things
DCC	digital calibration certificate
MI	measuring instruments
PoW	Proof-of-Work
PoS	Proof-of-Stake
PoA	Proof-of-Authority
LR	legally relevant
NLR	legally non-relevant
PKI	public-key infrastructure
NMI	national metrology institute

## Appendix A. References Relevant to the State of the Art

There are numerous papers on IoT blockchain applications and the digital transformation of metrology. We have categorized all referenced papers according to the six most relevant topics for our paper (Table A1). Thereby, references from IB and OB categories also belong to the B category, but references were not repeated in the latter due to space optimization. In the same way, TM is a subcategory of M, and BTM is a subcategory of TM and B. For the category “Miscellaneous”, i.e., references that do not have a focus on of our most relevant six categories, the main topic is listed in the table.

**Table A1:** Categories of referenced papers.

Blockchain in general (B)		[1,2,16,17,18,19,26,37,38]
IoT blockchain application (IB)		[10,11,12,27]
Oracle issue of blockchain (OB)		[3,23,25]
Metrology in general (M)		[4,5,22,28,33,44,47]
Digital transformation of metrology (TM)		[6,7,8,9,20,21,42,43]
Blockchain as possible infrastructure for digital transformation of metrology (BTM)		[13,14,15,24,45,46]
Miscellaneous	anti-tampering and security	[29,30,31,32,33]
	multi-sensor approach	[35,36]
	data market	[39,40,41]

Although important for each particular use case, no reference has a holistic approach, and this kind of approach is crucial if one wants to establish a full trust hierarchy as explained in more detail in Section 2, and thoroughly analyzed throughout our paper, which is trying to close precisely this gap.

## Appendix B. Blockchain Platforms and WELMEC Regulations

Table A2 shows which criteria of WELMEC regulations can be fulfilled by Ethereum and Hyperledger Fabric.

**Table A2:** Blockchain platforms and WELMEC regulations.

	Ethereum	Hyperledger Fabric
L1. Completeness of measurement data stored	yes	yes
L2. Protection against accidental or unintentional changes	no	yes
L3. Integrity of data	yes	partial
L4. Traceability of stored measurement data	yes	yes
L5. Confidentiality of keys	yes	yes
L6. Retrieval, verification, and an indication of stored measurement data	yes	yes
L7. Automatic storing	no	no
L8. Storage capacity and continuity	yes	yes
T1. Completeness of transmitted data	yes	yes
T2. Protection against accidental or unintentional changes	no	yes
T3. Integrity of data	yes	partial
T4. Traceability of transmitted measurement data	yes	yes
T5. Confidentiality of keys	yes	yes
T6. Receiving, verification and handling of transmitted measurement data	yes	yes
T7. Availability of transmission services	yes	yes
T8. Transmission delay	yes	yes
S1. Realization of software separation	partial	partial
S2. Mixed indication	yes	yes
S3. Protective software interface	yes	yes
D1. Download mechanism	yes	yes
D2. Authentication of transmitted software	yes	yes
D3. Integrity of downloaded software	yes	yes
D4. Traceability of legally relevant software download	yes	yes
